# Molecular detection and identification of *Babesia bovis* and *Trypanosoma* spp. in one-humped camel (*Camelus dromedarius*) breeds in Egypt

**DOI:** 10.14202/vetworld.2021.625-633

**Published:** 2021-03-12

**Authors:** Shimaa Abd El-Salam El-Sayed, Mohamed A. El-Adl, Mayar O. Ali, Mostafa Al-Araby, Mosaab A. Omar, Mohamed El-Beskawy, Shimaa Sobhy Sorour, Mohamed Abdo Rizk, Magdy Elgioushy

**Affiliations:** 1 Department of Biochemistry and Chemistry of Nutrition, Faculty of Veterinary Medicine, Mansoura University, Mansoura 35516, Egypt; 2 National Research Center for Protozoan Diseases, Obihiro University of Agriculture and Veterinary Medicine, Inada-Cho, Obihiro, Hokkaido, Japan; 3 Department of Animal Genetics, Faculty of Veterinary Medicine, Mansoura University, Mansoura 35516, Egypt; 4 Department of Parasitology, Faculty of Veterinary Medicine, Mansoura University, Mansoura 35516, Egypt; 5 Department of Veterinary Medicine, College of Agriculture and Veterinary Medicine, Qassim University, 51452 Qassim, Saudi Arabia; 6 Department of Parasitology, Faculty of Veterinary Medicine, South Valley University, 83523, Qena, Egypt; 7 Animal Medicine Department (infectious diseases), Faculty of Veterinary Medicine, Matrouh University, Egypt; 8 Department of Parasitology, Faculty of Veterinary Medicine, Kafrelsheikh University, 33516, Kafrelsheikh, Egypt; 9 Department of Internal Medicine and Infectious Diseases, Faculty of Veterinary Medicine, Mansoura University, Mansoura 35516, Egypt; 10 Department of Animal Medicine, Faculty of Veterinary Medicine, Aswan University, Aswan, 37916, Egypt

**Keywords:** *Babesia bovis*, camel, Egypt, epidemiology, *Trypanosoma* spp

## Abstract

**Background and Aim:**

Camels are a unique source of milk and meat, which helps recover from several diseases that affect humans worldwide. In Egypt, one of the great obstacles for this industry is tick-borne diseases. This study aimed to characterize blood parasite infections, such as *Babesia* (*B*.) *bovis* and *Trypanosoma* (*T*.) spp. in one-humped camel (*Camelus dromedarius*) (n=142) breeds in Halayeb and Shalateen, Egypt, through phylogenetic analysis.

**Materials and Methods:**

The prevalence of *B. bovis* and *Trypanosoma* spp. was identified in camels using polymerase chain reaction (PCR) assays targeting the *Rhoptry-Associated Protein-1* and *internal transcribed spacer 1* genes, respectively. A nested PCR technique was conducted to detect *B. bovis*. At the same time, KIN multispecies PCR assay was employed to diagnose and classify trypanosome DNA in camels.

**Results:**

*B. bovis* was detected in 4/142 camels with an infection rate of 2.81%. Sequencing and phylogenetic analyses revealed that the strain of *B. bovis* isolated from this population was closely related to strains isolated from Argentine, the United States, and Brazil. Moreover, *Trypanosoma evansi* was detected in 8/142 camels with an infection rate of 5.63%. Sequencing and phylogenetic analyses revealed that this isolated strain *T. evansi* was closely related to *Trypanosoma theileri* detected from cattle in Brazil.

**Conclusion:**

The obtained data indicated the existence of *B. bovis* and *T. evansi* in camels from two provinces of Egypt. The obtained findings have economic significance and reflect the importance of implementing effective prevention and control methods across Egypt to reduce the incidence of *B. bovis* and *T. evansi* in camels.

## Introduction

Global concerns about desertification and the resulting loss of biological productivity due to natural processes, climate change, or human activities have highlighted the important role of camels in socioeconomic aspects to protect human populations from health risks and promote species conservation. Thus, it is critical to implement a proper management system with appropriate disease control measures [1]. In several regions of the world, camels provide an important source of food and milk, which is considered a high protein source with substantial nutritive value. Moreover, the unique survival capability of camels in adverse environmental conditions permits breeders to adopt semi-intensive farming systems for camel production [2]. It was thought that camels are resistant to various pathogenic diseases [3]. Recently, different literatures have described the susceptibility of camels to large number of pathogenic agents, such as bacteria, fungi, parasites, and viruses [4].

In Egypt, vector-transmitted diseases cause various clinical manifestations in farm animals [3]. Members of piroplasms have been found in several species of equines, cattle, and camel in Egypt, where specific DNA fragments of those piroplasms were detected in the bloodstream of apparently healthy camels of *Babesia* and *Theileria* species [4]. One well-known piroplasm is *Babesia* spp., which infects humans through tick infestation [5]. Camel babesiosis is an acute to chronic infectious disease that is distributed all over the world [2]. The disease is responsible for deteriorative effects, high morbidity, and substantial economic losses due to transmission through tick-borne hemoparasitic protozoa [2,4]. *Trypanosoma evansi* is a well-known parasite that affects animals and is transmitted mechanically by tabanid species. *T. evansi* infections cause weight loss, anemia, and abortion in populations spanning several regions of the world, such as South America, Asia, and Africa [6]. In certain cases of trypanosomiasis, subclinical cases and other chronic infections can be found in hosts infected by low virulence strains [7].

Veterinary epidemiology offers the means to investigate disease outbreaks by tracking and monitoring diseases, identifying disease risk factors, implementing herd health programs, and developing biosecurity measures [8]. Accumulating epidemiological knowledge will lead to innovative strategies and clinical techniques that will result in the reduction, prevention, and eradication of important infectious diseases in animals as blood parasitic infections [9]. In this regard, a molecular technique using a polymerase chain reaction (PCR) assay with sequencing analysis can identify blood parasites in epidemiological studies with higher accuracy than other traditionally diagnostic methods, such as microscopic examination of Giemsa-stained blood smear and enzyme-linked immunoassay (ELISA) tests [3]. The latter two diagnostic tests (microscopy and ELISA) have low sensitivity against chronic and subclinical infections [10], with a lack of specificity in distinguishing between recent and old infections [7]. Most previous methods for epidemiological screening of blood parasite in camels that were performed in Egypt [11-14] used ELISA, the microscopy method, or PCR without performing sequencing and phylogenetic analysis for the diagnosis of parasites. Moreover, most of the previous studies screened only one blood parasite. For the last few years, most of the camels imported to Egypt came from Sudan. Identification and phylogenetic analysis of blood parasites in camels raised on the border between Egypt and Sudan are of great economic importance.

Therefore, this study aimed to use molecular diagnostic techniques to detect parasitic blood infections (*Babesia bovis* and *Trypanosoma* spp.) in camel breeds in Halayeb and Shalateen in Egypt followed by phylogenetic analysis to identify each parasite.

## Materials and Methods

### Ethical approval and informed consent

In this study, all experimental protocols were approved by the Animal Care and Use Committee, Faculty of Veterinary Medicine, Mansoura University, Egypt. According to the Egyptian Medical Research Ethics Committee (No. 14-126), all Institutional and National Guidelines for the care and use of animals were followed. All experiments were carried out under the authority of the Ministry of Education, Egypt, in compliance with the Fundamental Guidelines for Proper Conduct of Animal Experiment and Related Activities in Academic Research Institutions. Informed written consent was obtained from the owner.

### Study period and location

The screened blood samples were collected during July 2017 from one-humped camels reared in Halayeb and Shalateen in Egypt, at the Sudan border. The samples were processed at Faculty of Veterinary Medicine, Mansoura University, Egypt.

### Sampling

**Figure-1:**
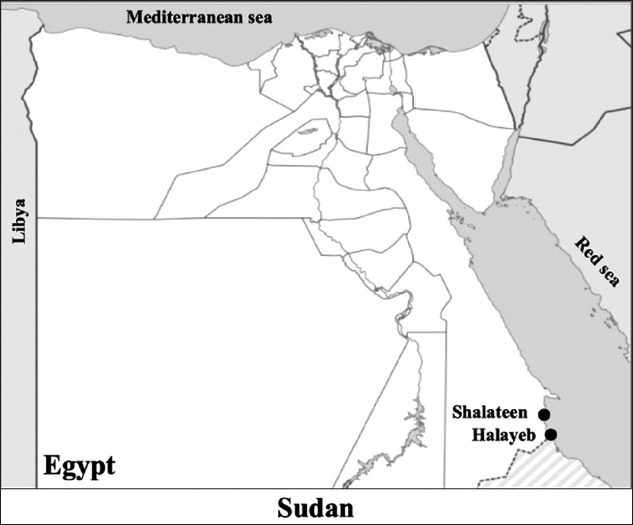
Map of the study sites of the blood parasites molecular epidemiology study in Egypt. Blood samples were collected from animals reared in Halayeb and Shalateen as indicated by bullet points [Source: https://simplemaps.com/resources/svg-eg].

One hundred and forty two blood samples were collected from one-humped camels reared in Halayeb and Shalateen in Egypt, at the Sudan border (Figure-1). All obtained blood samples were tested for infection with *B. bovis* and *Trypanosoma* spp. During the sampling period, all animals were apparently healthy.

### DNA extraction and PCR detection of hemoparasites

Blood samples were collected from the jugular veins of camels selected for our study. Approximately 2 mL whole blood was collected from each animal in a Vacutainer tube containing ethylenediaminetetraacetic acid (EDTA). The blood samples were labeled and stored at −20°C until DNA extractions were conducted. The DNA samples were extracted from 300 μL blood samples using a commercial kit (Promega, Madison, WI, USA), following the manufacturer’s instructions, and then stored at −20°C until further use. *B. bovis* and *Trypanosoma* spp. were detected in DNA samples using a previously described diagnostic PCR assay targeting *Rhoptry-Associated Protein-1* (*RAP-1*) and *internal transcribed spacer 1* (*ITS1*) genes, respectively [15-17]. Nested PCR assays were used for the detection of *B. bovis*. KIN multispecies PCR reaction was employed to detect and identify trypanosome DNA in camels. A KIN multispecies PCR amplifies *ITS1* and simultaneously detects three major trypanosome species: *T. evansi*, *Trypanosoma congolense*, and *Trypanosoma vivax* [16]. Primer sequences and annealing temperatures are shown in Table-1. Enzyme activation and denaturation used for the amplification conditions for *B. bovis* and *Trypanosoma* spp. were 95°C for 3 min and 95°C for 30 s, 95°C for 5 min and 95°C for 30 s, and 94°C for 3 min and 94°C for 1 min, respectively [15-17]. After the product was chilled to 4°C, gel electrophoresis of the PCR products was performed on 1.5% agarose gel with Tris/Borate/EDTA buffer and was stained with ethidium bromide. The final PCR product was then visualized under ultraviolet light.

**Table-1:** Primary and nested PCR primers used for PCR amplifications.

Species	Assay	Primer sequence (5′ → 3′)	Annealing	Amplification cycles (No.)	Product size	Target gene	References
*B. bovis*	PCR	F-CACGAGCAAGGAACTACCGATGTTGA R- CCAAGGACCTTCAACGTACGAGGTCA	55°C	45	360 bp	*RAP-1*	[15-17]
	nPCR	F- TCAACAACGTACTCTATATGGCTACC R- CTACCGACCAGAACCTTCTTCACCAT	55°C	35	298 bp		
*Trypanosoma* spp*.*	KIN-PCR	F- GCGTTCAAAGATTGGGCAAT R- CGCCCGAAAGTTCACC	60°C	40	540 bp	*ITS1*	[16]

*B. bovis*: *Babesia bovis*, PCR=Polymerase chain reaction, *RAP-1*=*Rhoptry-Associated Protein-1*, *ITS1*=*Internal transcribed spacer 1*

### Cloning and sequencing of PCR products

Extraction of amplicons of PCR samples with high band intensities using a QIAquick Gel Extraction Kit (QIAGEN, Hilden, Germany) was conducted from agarose gels. The samples were then cloned into a plasmid vector (PCR 2.1-TOPO, Invitrogen, Carlsbad, CA, USA). A genetic analyzer AB1 PRISM 100 (Applied Biosystems, Foster City, CA, USA) was used to sequence two colonies for each amplicon.

### Phylogenic analysis

The evolutionary history was predicted using the neighbor-joining method [18]. The optimal tree with a sum of 3-branch length = 56.08480007 is shown. The percentage of replicate trees in which associated taxa clustered together in the bootstrap test (1000 replicates) is shown next to the branches [19]. Evolutionary distances were computed using the maximum composite likelihood method [20] and are represented by the units of the number of base substitutions per site. The analysis involved 17 nucleotide sequences. The codon positions were 1^st^+2^nd^+3^rd^+Noncoding. All positions containing gaps and missing data were eliminated. There were 201 positions in the final dataset. Evolutionary analyses were conducted using MEGA6 [20].

### Statistical analysis

The Open Epi program was used to detect the upper and lower limits of the confidence intervals of the positive rates for *B. bovis* and *T. evansi* parasites (http://www.openepi.com/v37/Proportion/Proportion.htm).

## Results

**Supplementary Figure-1:**
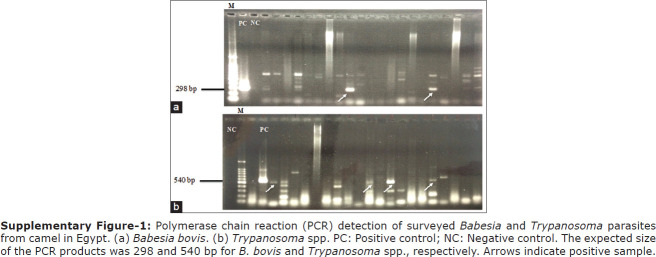
Polymerase chain reaction (PCR) detection of surveyed *Babesia* and *Trypanosoma* parasites from camel in Egypt. (a) *Babesia bovis*. (b) *Trypanosoma* spp. PC: Positive control; NC: Negative control. The expected size of the PCR products was 298 and 540 bp for *B. bovis* and *Trypanosoma* spp., respectively. Arrows indicate positive sample.

The genomic DNA sequences of the detected blood parasites in camel blood were subjected to PCR with universal primers to detect all possible strains of *Babesia* and *Trypanosoma* species to amplify small ribosomal subunits in those parasites using a molecular diagnostic approach. The infection rates for *B. bovis* and *T. evansi* were 2.81% and 5.63%, respectively (Table-2). Species-specific PCR assays detected *B. bovis* and KIN multispecies, while the PCR detected *Trypanosoma* species in the camel populations (Supplementary Figure-1). One sample harbored mixed infections from *Babesia* and *Trypanosoma* parasite species.

**Table-2:** PCR detection of blood parasites in camels.

Species	Positive No.	% CI^a^
*B. bovis*	4	2.81 (1.10 7.01)
*T. evansi*	8	5.63 (2.88 10.72)

a 95% confidence interval, *B. bovis*: *Babesia bovis*, PCR=Polymerase chain reaction, *T. evansi=*T*rypanosoma evansi*

The nucleotide sequences of PCR amplified *RAP-1* and *ss-rRNA* genes of *Babesia* species and the *ITS1* gene of *Trypanosoma* species determined in this study have been registered and were assigned the following GenBank accession numbers: *B. bovis* (MF737083.1) and *T. evansi* (MF737081.1). For phylogenetic analysis, nucleotide sequences of the target genes from other species of *Babesia* and *Trypanosoma* were included for comparison (Tables-S1 and S2).

**Table-S1:** *Babesia* and *Trypanosoma* species used for genetic analysis in this study.

Babesia species	Origin		Accession number

	Geographic	Biological	
*B. bovis*	Uruguay	Cattle	AF030061
	North Brazil	Cattle	FJ588011.1
	China	Cattle	KT318580
	USA	Cattle	AF030053.1
	Argentina	Cattle	AF030055.1
	Texas USA	Cattle	AF030059.1
	Mexico	Cattle	AF027149.1
	South Africa	Bovine	KC894392.1
	Philippines	Cattle	LC006976.1
	Colombia	Cattle	KX365053.1
	Brazil	Bovine	JX177355.1
	China	Cattle	KT312807.1
	China	Cattle	KT318580.1
	China	Cattle	KT312814.1
	Cuba	Buffalo	JF279443.1
	Sri Lanka	Buffalo	AB845432.1
	Egypt	Cattle	AB917246.1
	China	Cattle	KT312810.1
	Guinea-Bissau	Cattle	KC312810.1
	Brazil	Cattle	KC964615.1
	**Egypt**	**Camel**	**MF737083.1^a^**
	Argentine	Cattle	AF030056.2
	USA	Cattle	AF030054.1
	Egypt	Cattle	BAP47555.1
	China	Cattle	ALJ26466.1
	USA	Cattle	AAB84269.1
	USA	Cattle	AAB84264.1
	Mexico	Cattle	AAC27387.1
	USA	Cattle	AAB84263.1
	China	Cattle	AKR16131.1
	Philippines	Cattle	BAP81796.1
	Argentine	Cattle	AAB84266.1
	China	Cattle	AKR16127.1
	Sri Lanka	Buffalo	BAO50704.1
	China	Cattle	AKR16124.1
	Brazil	Buffalo	AGT18676.1
	Colombia	Cattle	APP91268.1
	Argentine	Cattle	AAB84265.1
	USA	Cattle	AAB84264.1
	Portugal	Cattle	ACY76255.1
	Sri Lanka	Cattle	BAW18785.1
	South Africa	Bovine	AGU67927.1
	Uruguay	Cattle	AAB84271.1
	Brazil	Cattle	ACM44004.1
	Cuba	Buffalo	AEI52891.1
*B. bovis*	Brazil	Bovine	AFQ30755.1
	Germany	Vole	AB085191.1

a GenBank accession numbers submitted by this study. *B. bovis*: *Babesia bovis*

**Table-S2:** *Trypanosoma* species used for genetic analysis in this study.

Trypanosoma species	Origin		Accession number

	Geographic	Geographic	
*Trypanosoma microti*	Field vole	United Kingdom	AY043354.1
*Trypanosoma minasense*	Squirrel-monkeys	Japan	AB362412.1
*Trypanosoma grosi*	Wood mouse	United Kingdom	AY043355.1
*Trypanosoma evotomys*	Bank vole	United Kingdom	AY043356.1
*Trypanosoma herthameyeri*	Criolla frog	Brazil	KT765860.1
*Trypanosoma teixeirae*	Red flying fox	Australia	KT907061.1
*Trypanosoma cruzi*	ND	USA	LT220268.1
*Trypanosoma livingstonei*	Roundleaf bat	Mozambique	KF192994.1
*Trypanosoma rangeli*	bugs	Brazil	KT368799.1
***T. evansi***	**Camel**	**Egypt**	**Mf737081.1^a^**
*T. theileri*	Cattle	Brazil	AY773713.1
*Trypanosoma dionisii*	Free-tailed bat	Brazil	JN040980.1
*T. vivax*	ND	Brazil	AY363165.1
*Trypanosoma equiperdum*	Horse	South Africa	KY609969.1

a GenBank accession numbers submitted by this study. *T. evansi*=T*rypanosoma evansi*, *T. theileri*=*Trypanosoma theileri*, *T. vivax*=*Trypanosoma vivax*

**Figure-2:**
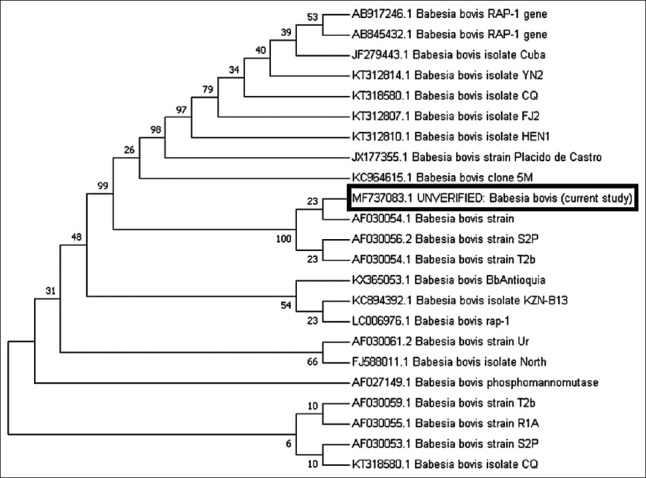
Phylogenic tree of *Babesia bovis* *Rhoptry-Associated Protein-1* gene. The nucleotide sequences determined in this study are shown in boldface type letters. Bootstrap values are provided at the beginning of each branch. The sequence identified from Egypt in this study is boxed in black.

**Supplementary Figure-2:**
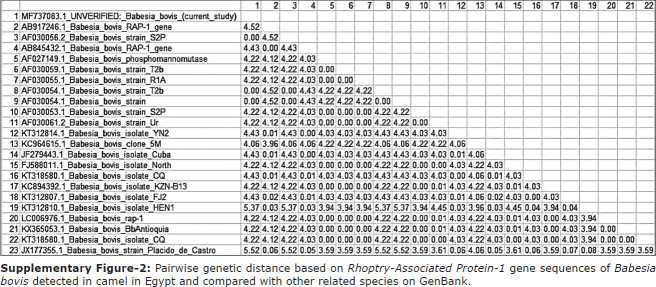
Pairwise genetic distance based on *Rhoptry-Associated Protein-1* gene sequences of *Babesia bovis* detected in camel in Egypt and compared with other related species on GenBank.

**Figure-3:**
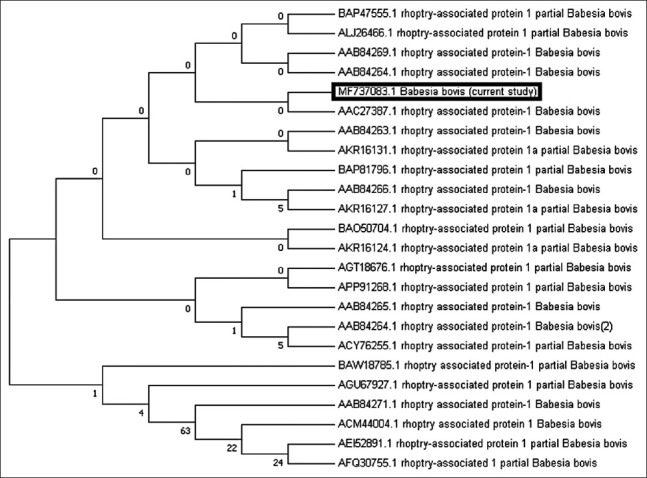
Protein’s phylogenetic tree of *Babesia bovis* *Rhoptry-Associated Protein-1* gene. The amino acids sequences determined in this study are shown in boldface type letters. Bootstrap values are provided at the beginning of each branch. The sequence identified from Egypt in this study is boxed in black.

**Supplementary Figure-3:**
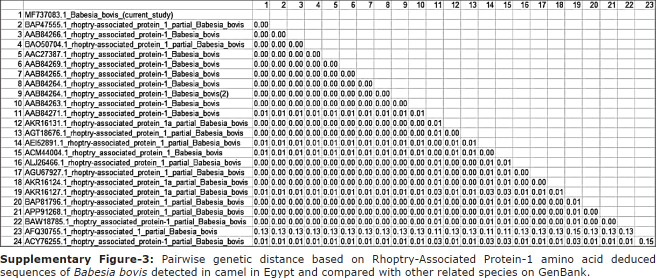
Pairwise genetic distance based on Rhoptry-Associated Protein-1 amino acid deduced sequences of *Babesia bovis* detected in camel in Egypt and compared with other related species on GenBank.

Detection of *B. bovis* in camel blood was determined using primer pairs flanking *RAP-1*. PCR of *RAP-1* produced an amplicon size of 298 bp that was evaluated by GenBank for further confirmation of the determined sequence. The confirmed sequence was deposited in GenBank under accession number MF737083.1, where a similarity index test among other related *B. bovis* species was conducted using GenBank. A phylogenetic analysis was constructed using our obtained species and other related sequences of the *RAP-1* gene in *B. bovis*. The phylogenetic analysis revealed a significant decrease in genetic distance between our obtained species and other species isolated from Argentina (AF030056), the United States (AF030054), and Brazil (KC964615) (Figure-2). Interspecies genetic distance analysis was conducted between our obtained *RAP-1* gene sequences and sequences of related taxa, and a close genetic distance was detected between our isolated strain and other taxa isolated from cattle in Argentina (AF030056.2) and the United States (AF030054.1) (Supplementary Figure-2). For further confirmation, an amino acid sequence was deduced from our obtained sequence and was aligned with other related sequences of *RAP-1* protein (Supplementary Figure-2). The results of the phylogenetic analysis of proteins showed a close genetic relationship with other strains from Mexico (AAC27387.1) (Figure-3). Pairwise genetic distance was performed between our obtained amino acid sequence and sequences of related species of *RAP-1* genes, and close relationships with all aligned species except the isolated species from Brazilian bovine species (AFQ30755.1) were revealed (Supplementary Figure-3).

**Figure-4:**
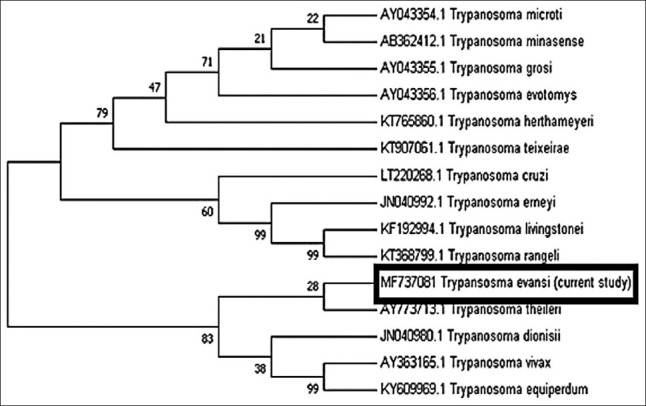
Phylogenic tree of *Trypanosoma evansi internal transcribed spacer 1* gene. The nucleotide sequences determined in this study are shown in boldface type letters. Bootstrap values are provided at the beginning of each branch. The sequence identified from Egypt in this study is boxed in black.

**Supplementary Figure-4:**
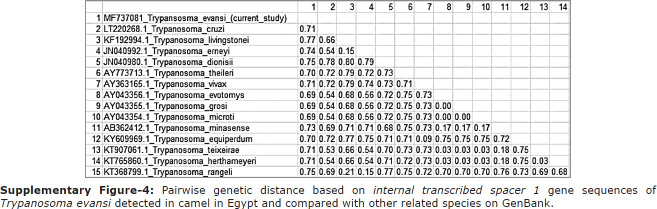
Pairwise genetic distance based on *internal transcribed spacer 1* gene sequences of *Trypanosoma evansi* detected in camel in Egypt and compared with other related species on GenBank.

Internal transcript spacer 1 was used for simultaneous identification of three major trypanosome species (*T. evansi*, *T. congolense*, and *T. vivax*) in camels reared in Upper Egypt, with a product size of 540 bp using a KIN multispecies PCR assay. The sequencing results indicated that *T. evansi* is the causative agent of trypanosomosis in the camels under study. An accession number was deposited in GenBank for our obtained sequence (MF737081) (Figure-4). A phylogenetic tree was constructed depicting our isolated species of *T. evansi* and with related species where a higher similarity index was aligned with our sequence, and a lower genetic distance was observed between our isolated sequence and *Trypanosoma theileri* isolated from cattle in Brazil. The phylogenetic analysis revealed the presence of two clades (mammalian and non-mammalian), wherein *T. evansi* clustered with the mammalian group (Figure-4). Genetic distance analysis of our isolated *T. evansi* and other related trypanosome species revealed close genetic distance with AY0433156.1 from the United Kingdom in bank vole, AY043355.1 from the United Kingdom in wood mouse, and AY043354.1 from the United Kingdom in field vole with a 0.69 value in comparison with other included taxa (Supplementary Figure-4).

## Discussion

A phylogenetic analysis was designed for our isolated sequence and other sequences with a high similarity index using the neighbor-joining method for statistical and phylogenetic analysis of 500 replicates. A close genetic relationship was observed between our strain and other strains from Argentina, the United States, and Brazil. A similar result was investigated through research conducted by Mtshali *et al*. [21] who found that a nested PCR technique to amplify *RAP-1* revealed a close genetic relationship of a South African strain of *B. bovis* strains with other strains from Uruguay, Argentina, Brazil, and the United States. However, the presence of polymorphism can adequately discriminate between two species [22]. Similarly, the conservation of *Babesia* spp. sequence among species was observed among Brazilian strains of *B. bovis* (98-100%) [23].

Three main trypanosome species (*T. evansi*, *T. congolense*, and *T. vivax*) can be simultaneously detected in the circulating blood of camels using internal transcript spacer 1. A genetic relationship between our isolated species and other *Trypanosoma* species has been established through phylogenetic analysis. In this study, *T. evansi* was identified through PCR sequencing and was determined to be closely related to *T. theileri* detected from cattle in Brazil. *Trypanosome* was classified into several clades in a study in 1996 by Maslov *et al*. [24] which are as follows: Mammalian, bird group, and elasmobranch, vertebrates, and invertebrates parasites. Hughes and Piontkivska [25] classified trypanosome species according to the amino acid sequence of a small ribosomal subunit into 42 protein families. They also classified *Trypanosoma* families according to the host and location into American, and African. In Egypt, internal transcript spacer 1 was used to investigate the phylogenetic relationship among trypanosome species and revealed genetic diversity among those species, which can be used as a diagnostic tool for the identification of trypanosome species [26].

This study has some limitations that should be noted. The small sample size used in this study may not allow for a generalizable conclusion about the incidence rate of different hemoparasites included in the study. Moreover, this study was carried out in Upper Egypt, which so further investigation is needed to determine the presence blood parasites in other areas of Egypt.

## Conclusion

The data provided here indicate the presence of *B. bovis and T. evansi* in camels from two provinces of Egypt. These findings are of economic significance and reflect the importance of adopting successful prevention and control methods across Egypt to reduce the incidence and spread of blood parasite infection in camels.

## Authors’ Contributions

SAEE, MAR, MAE, and ME: Conceived and designed the experiments. SAEE, MAR, MAE, MOA, and ME: Performed the experiments. SAEE, MAE, MAO, and ME: Analyzed the data. SAEE, MAR, MAE, MA, ME, ME, and SSS: Contributed reagents/materials/analysis tools. SAEE, MAR, MAE, and ME: Wrote the manuscript. All authors reviewed the manuscript.

### Data Availability

All data collected during this study are included in this article.

### Competing Interests

The authors declare that they have no competing interests.

### Publisher’s Note

Veterinary World remains neutral with regard to jurisdictional claims in published map and institutional affiliation.
